# Effects of myotherapy combined with photobiomodulation on the lips: a randomized clinical trial

**DOI:** 10.1590/2317-1782/e20240144en

**Published:** 2025-08-08

**Authors:** Mariana Rodrigues Batista, Andréa Rodrigues Motta, Renata Maria Moreira Moraes Furlan

**Affiliations:** 1 Programa de Pós-graduação em Ciências Fonoaudiológicas, Universidade Federal de Minas Gerais – UFMG - Belo Horizonte (MG), Brasil.; 2 Departamento de Fonoaudiologia, Faculdade de Medicina, Universidade Federal de Minas Gerais – UFMG - Belo Horizonte (MG), Brasil.

**Keywords:** Lip, Muscle Strength, Muscle Tone, Laser Therapy, Myofunctional Therapy

## Abstract

**Purpose:**

to compare the maximum pressure, average pressure, and labial resistance of healthy adults undergoing myotherapy combined with photobiomodulation at different doses.

**Methods:**

a randomized, double-blind clinical trial was conducted. The non-probabilistic sample consisted of 12 individuals with a mean age of 21.8 years, randomly assigned to three distinct groups for intervention with photobiomodulation. The exercises were the same for all participants. In group 1 (G1), participants received photobiomodulation at a dose of 7 J per point; in group 2 (G2), participants received 9 J per point; and in the placebo group (PG), participants underwent the same procedures as in the other groups, but the device was turned on without being activated (placebo). The wavelength used was infrared. Participants received photobiomodulation three times a week, with a 48-hour interval, for eight weeks. Myotherapy was performed at home by participants three times a day, five times a week, for eight weeks. Clinical assessment of the lips, maximum pressure, average pressure, and labial resistance were conducted using the Iowa Oral Performance Instrument (IOPI) before and after intervention.

**Results:**

There were no results indicating an increase in maximum or average lip pressure; however, there was an increase in labial resistance in G1.

**Conclusion:**

Photobiomodulation associated with myotherapy, with the parameters and methodology used in this study, did not result in an increase in lip pressure but promoted an increase in resistance in the group exposed to 7 J per point

## INTRODUCTION

Laser is an English word that stands for light amplification by stimulated emission of radiation^(1)^. Photobiomodulation (PBM) with low-intensity laser is a medication-free, painless, non-invasive treatment method with no side effects^(1)^. Its benefits include improved muscle performance, delayed fatigue, muscle relaxation, increased strength gain, pain relief, reduced inflammation, and stimulation of tissue regeneration^(1-7)^.

These results are due to human cells being functional units that can be activated by photochemical, photophysical, and photobiological effects when tissues are irradiated^(8)^. Low-intensity laser is a type of non-ionizing radiation that stimulates the cells without destructive effects^(9)^. When entering biological tissue, the light can transfer its energy to that medium. Absorption depends on the action of chromophores – i.e., molecules present mainly in mitochondria that convert light energy into chemical energy during cellular metabolism^(10)^. The laser increases the amount of adenosine triphosphate (ATP) within cells through enzymatic reactions^(10)^.

PBM has been associated with the training of different muscles, especially large groups. There is little evidence to support the use of the resource^(11-13)^ for smaller muscle groups such as the orofacial muscles. Nevertheless, speech-language-hearing pathologists report applying PBM in their clinical practice^(14)^.

Myotherapy, a therapeutic approach present in clinical practice with oral-motor function, aims to modify muscles through exercises directed at the muscles it intends to stimulate^(15)^. Knowing that muscle activity requires a large amount of energy for contraction, relaxation, and maintenance of body tone^(16)^ and that PBM with low-intensity laser increases ATP within cells^(9)^, it is believed that PBM has the potential to accelerate muscle modification and optimize therapy time.

A study investigated the effects of PBM on the anterior thigh muscles and found that participants who were irradiated before muscle training had a significant increase in maximum voluntary contraction (MVC), besides the positive effect on the maximum repetition test^(5)^.

Regarding the effects of PBM on the lips, Mouffron et al.^(12)^ found an increase in lip pressure immediately after irradiation with an infrared wavelength laser and 7 J of energy. Batista et al.^(13)^, in turn, found no change in the electromyographic fatigue of the orbicularis oris due to irradiation with a dose of 4 J. No studies with more than one PBM session or combining muscle training with irradiation were found in the literature. It is believed that the higher the dose, the greater the photochemical effect, and, therefore, better muscle performance.

Thus, this study aimed to verify whether PBM associated with myotherapy enhances the gain in pressure and resistance of the lips in healthy adults.

## METHODS

This is a randomized, double-blind clinical trial conducted at Universidade Vale do Rio Doce (UNIVALE), Brazil. The study was approved by the institution's Research Ethics Committee (approval no. 6,237,793), registered on the Brazilian Clinical Trials Registry (ReBEC) platform (RBR-6pygc5m), and developed following the Consort^(17)^ Checklist (Appendix A). All participants signed an informed consent form. Blinding was as follows: the researcher who assessed the participants did not know which intervention group each one belonged to. The researcher who performed the intervention did not know the assessment results, and neither did the participants know what dose they were being irradiated with. Data were collected between August and December 2023. These are preliminary data from still ongoing research.

The selected sample was non-probabilistic, comprising 12 individuals, all UNIVALE students, in Governador Valadares, with a mean age of 21.8 years (SD = 4.0; minimum = 18; maximum = 28), all females. They were recruited through announcements during classes and dissemination on social media.

Participants were randomly divided into three groups by drawing lots: group 1 (G1), group 2 (G2), and placebo group (PG). To do so, participants took a piece of paper with a number from inside a box.

The study included healthy individuals aged 18 to 35 years who had not used muscle relaxant and/or anti-inflammatory medication^(18)^ in the 48 hours before data collection, who were not undergoing orofacial myofunctional therapy, and could perform the sustained lip protrusion exercise. It excluded participants who abandoned the study and did not return for reassessment; who had contraindications for phototherapy, according to the equipment manufacturers' manual (photosensitivity, pregnancy, glaucoma, undiagnosed lesion on or near the area to be irradiated, infection at the application site, history of cancer, and use of a pacemaker or other electronic implant).

### Procedures

**Figure 1:**
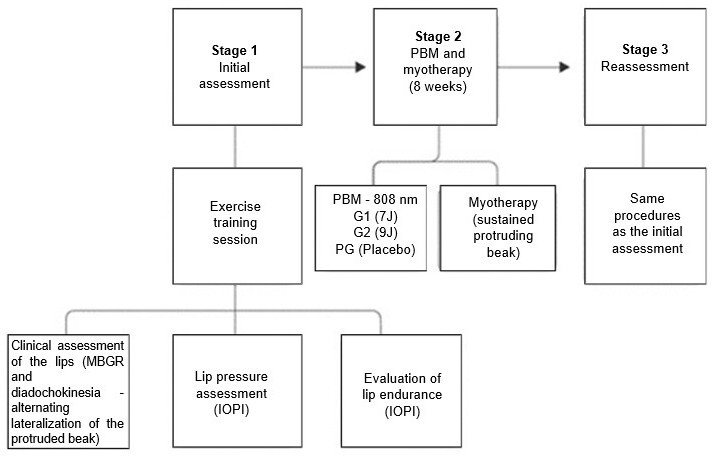
Assessment, intervention and reassessment procedures

The evaluation and PBM procedures were performed at the Oral-Motor Function Outpatient Clinic of UNIVALE’s Integrated Clinics. The procedures were divided into three stages: an initial evaluation session (stage 1), 8 weeks of individual treatment (stage 2), and a final reevaluation session (stage 3), as exemplified in Figure 1.

#### Stage 1 - Evaluation

Stage 1 began with an instruction session on how to perform the exercise correctly, followed by verification of the participant's ability to perform it. Then, the initial assessment was carried out, in which the participants underwent clinical evaluation of the lips, lip pressure, and lip resistance.

Evaluation and low-intensity-laser PBM were performed by different researchers, thus ensuring that the evaluator did not know about the participants’ allocation into specific groups.

To ensure an impartial data analysis, all evaluation files were numbered, and the participants’ names were removed, thus avoiding the identification of the groups to which they belonged during the analysis (whether before or after training).

##### Clinical evaluation of the lips

The participant was instructed to remain seated upright in a chair, with 90º flexion between hips, knees, and ankles, guided by the Frankfurt Plane. The sample was characterized through intraoral, extraoral, and lip mobility assessments, performed in accordance with the recommendations of the mouth-gastric-respiratory motor function orofacial myofunctional assessment (MBGR, in Portuguese)^(19)^.

##### Lip pressure assessment

The Iowa Oral Performance Instrument (IOPI) was used to obtain the lip pressure per participant. The IOPI bulb was placed between two wooden spatulas and wrapped in plastic film^(12,20,21)^. The participants were asked to press the bulb with maximum force, with a lip grip movement, for 2 seconds. Three repetitions were performed, with a 30-second interval between them, and the maximum peak value was considered the maximum lip pressure. The mean pressure (the arithmetic mean of the peak pressure obtained in each of the three repetitions) was also recorded.

##### Lip resistance assessment

The IOPI also obtained lip resistance values, with the bulb between two wooden spatulas and wrapped in plastic film. Participants were asked to press the bulb, maintaining 50% of the maximum pressure for as long as possible^(21)^. There was real-time visual feedback to indicate the pressure value that should be maintained. The time, in seconds, in which the participant was able to keep the bulb pressed was considered lip resistance.

#### Stage 2 – PBM and Myotherapy

At this stage, participants were randomly assigned to three groups by drawing lots. Participants did not know which group they were allocated to or which intervention they would receive. The researchers who analyzed the data also did not have access to which type of intervention each participant received, maintaining blindness until the end of the research.

- G1: subjected to myotherapy and PBM with an infrared wavelength and dose of 7 J per point, totaling 42 J.
- G2: subjected to myotherapy and PBM with an infrared wavelength and dose of 9 J per point, totaling 54 J.
- PG: subjected to myotherapy and placebo PBM procedure (the equipment was positioned and turned on, but there was no light emission).

**Figure 2:**
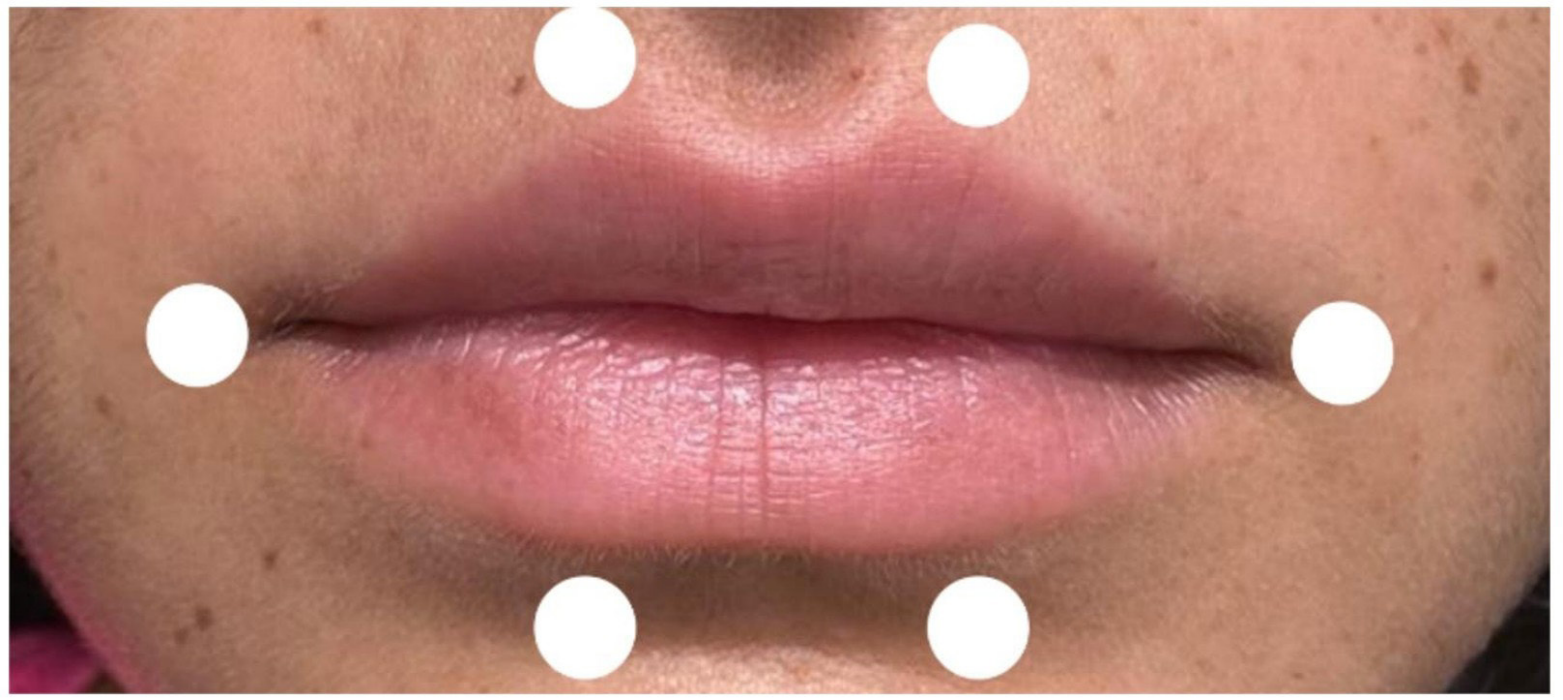
Marking the laser application points

Irradiation occurred through contact with the skin, with two points in the upper portion and two points in the lower portion of the orbicularis oris muscle, and one point at each corner of the mouth, as demonstrated in Figure 2.

##### Myotherapy

Myotherapy was performed in 8 weeks of treatment with home exercises three times a day for 5 days a week.

The therapeutic intervention was chosen based on findings in the literature regarding the type of exercise, repetitions, training frequency, and treatment duration. One study proposed to investigate and compare the electrical activity of the orbicularis oris muscle in different isometric maximum voluntary contraction exercises. It found that the lip protrusion exercise with closure presented greater electrical activity in the orbicularis oris muscle^(22)^.

Participants were instructed to perform three repetitions^(23,24)^ of the isometric lip protrusion exercise with closure^(22)^, three times a day^(24,25)^, 5 days a week^(26,27)^, for 8 weeks^(24,28)^. The proposed training had a progressive load, progressively increasing the time of exercise support every week, as shown in Chart 1.

**Chart 1:** Number of repetitions and contraction time of the lip protrusion exercise performed by participants each week of treatment

**Week**	**Repetitions**	**Contraction time (s)**
1	3	5
2	3	10
3	3	15
4	3	20
5	3	25
6	3	30
7	3	35
8	3	40

Each participant performed the exercise following a step-by-step guide and filled out a control table (Appendix B), sent by email and made available in Microsoft Word Online, according to their performance, including data such as the frequency of performance per day. They marked with an “X”, informing that they had performed the exercise in that shift, on the day in question.

##### PBM

After the initial assessment, laser applications were performed using MMO^®^ equipment, Laser Duo model, and 100 mW of power. Chart 2 shows the irradiation parameters.

**Chart 2:** Irradiation parameters

Irradiation parameters	Values
Wavelength	808 nm (infrared)
Mode of operation	Continuous
Power	100 mW
Output spot diameter	1.95 mm
Output spot area	0.03 cm^3^
Power density	3.3 W/cm^2^
Energy per point	7 J / 9 J
Energy density (fluence) per point	133.3 J/cm^3^
Application time per point	70 s / 90 s

The 808 nm (infrared)^(12,13)^ wavelength laser was chosen for irradiation, with the following dosimetric parameters: 7 J^(29-32)^ and 9 J per application point. A randomized clinical trial obtained immediate effects such as modification of lip pressure and improvement in lip muscle performance^(12)^, after PBM with a wavelength of 808 nm and a dose of 7 J.

Another study investigated the immediate effects of PBM on electromyographic fatigue of the orbicularis oris muscle, using a dose of 4 J per application point (two points in the upper portion and two points in the lower portion). It found no statistically significant differences between before and after irradiation^(13)^.

An integrative literature review^(33)^ indicated that the most used dosimetry per point was 7 J and 30 J. Considering the particularities of the orbicularis oris muscle, especially regarding thickness and extension, it was decided not to apply 30 J per point, but rather 9 J, which is the maximum dose available for a single (direct) application in the equipment. Six points were irradiated: one point at each corner of the mouth and two points at the upper portion and two at the lower portion of the orbicularis oris muscle.

The PBM was applied three times a week by previously trained researchers at the Oral-Motor Function Outpatient Clinic of UNIVALE’s Integrated Clinics, maintaining a minimum 48-hour interval between applications^(34)^. During the irradiation, the participants were seated, with their backs and feet supported, and wearing protective goggles. The procedure followed the recommendations of the equipment manufacturer and the safety standards established by the Brazilian National Health Surveillance Agency (ANVISA) for PBM equipment to ensure the applicators’ and participants’ safety.

The equipment was turned on for the placebo procedure, but with no light emission. The equipment was positioned with contact over the six application points for 70 to 90 s, equivalent to doses of 7 and 9 J, respectively, when irradiated.

#### Stage 3 - Reevaluation

Each participant was reevaluated by the same initial evaluator, using the same procedures as in the initial evaluation, the week following the last treatment session.

### Data analysis

This study’s response (outcomes) variables were mean and maximum lip pressure and lip resistance. The Shapiro-Wilk test was first applied, showing that the data did not have a normal distribution. The Kruskal-Wallis test verified homogeneity regarding age, maximum pressure, mean pressure, and resistance, measured before the intervention.

The Wilcoxon test - a non-parametric test for comparing two paired samples - was used to compare the mean maximum pressure and resistance between before and after the intervention.

**Figure 3:**
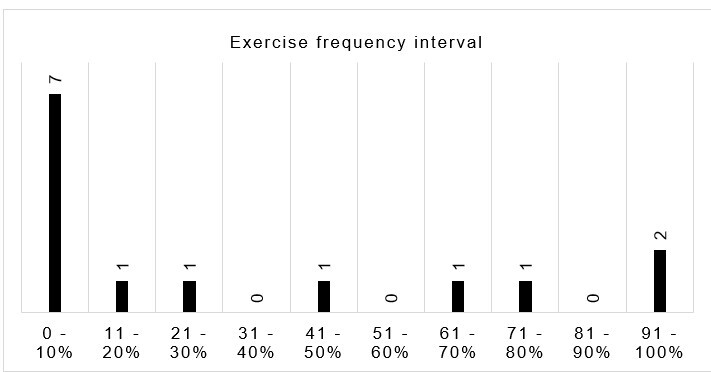
Frequency of exercise

The frequency with which participants performed the exercises over the 8 weeks was calculated based on the responses to the questionnaires and analyzed descriptively by relative frequency, as shown in Figure 3.

The differences in lip pressure and resistance from before to after the intervention were compared between individuals who performed the training at up to 50% and those who performed more than 50% of the stipulated frequency, using the Kruskal-Wallis test.

All comparisons used a 5% significance level.

## RESULTS

Initially, 29 participants were evaluated and included for data collection. However, only 12 participants remained until the final reevaluation stage. Fourteen participants were excluded after abandoning the study – five of these belonged to G1, three belonged to G2, three belonged to PG, and three did not receive any intervention and had not been allocated to any group. It was also decided to exclude another three male participants for data analysis since it was not possible to match the sample for sex.

The results indicated that the groups were homogeneous regarding age (p = 0.603), maximum pre-intervention pressure (p = 0.207), mean pre-intervention pressure (p = 0.149), and mean resistance (p = 0.059).

Table 1 shows the comparison of maximum and mean lip pressure and lip resistance before and after the intervention per group.

**Table 1:** Maximum pressure (kPa), mean pressure (kPa), and lip endurance before and after laser application

Group		Pre	Post	Pre	Post	Pre	Post
		(Max)	(Max)	(Mean)	(Mean)	(Endurance)	(Endurance)
G1	Mean	12.0	7.0	10.7	6	22.7	211.7
	SD	6.6	1.73	5.7	1.7	2.3	287.9
	Median	11.0	6	9	5	24	54
	Min	6	6	6	5	20	37
	Max	19	9	17	8	24	544
	p-value	0.246		0.121		0.0463*	
G2	Mean	7.6	9	6.7	8	91	53
	SD	0.6	2	0.6	2	38.5	38.5
	Median	8	9	7	7	90	54
	Min	7	7	6	6	53	14
	Max	8	11	7	10	130	91
	p-value	0.369		0.368		0.513	
PG	Mean	6.6	7.4	5.6	6.8	65.4	42.8
	SD	1.3	3.5	0.9	3.2	23.5	29.2
	Median	6	7	5	7	56	28
	Min	6	4	5	4	47	17
	Max	9	13	7	12	104	76
	p-value	0.914		0.747		0.347	

Wilcoxon test. Significance level of 5%

* p-value ≤ 0.05

**Caption:** G1 = group irradiated with 7 J per point; G2 = group irradiated with 9 J per point; PG = placebo group; SD = standard deviation

The comparative analysis of maximum and mean lip pressure before and after the participant underwent PBM did not indicate any statistically significant difference.

The comparative analysis of resistance between before and after the intervention indicated a difference with statistical relevance for G1, with increased resistance after the intervention.

Table 2 shows the comparison between the groups for the difference in maximum and mean lip pressure and lip resistance, calculated by subtracting the pre-intervention value from the post-intervention value.

**Table 2:** Difference in maximum pressure, mean pressure, and resistance among all groups

Group	Maximum pressure		Mean pressure		Endurance	
	mean	SD	Mean	SD	Mean	SD
G1	-5.0	5.0	-4.7	4.0	189	286.8
G2	1.3	2.5	1.3	2.0	-38	77.0
PG	0.8	4.4	1.2	3.8	-22.6	38.8
p-value^*^	0,282		0,186		0,234	

Kruskal-Wallis test. Significance level of 5%

Negative values indicate that the post-intervention value was lower than the pre-intervention value

* p-value ≤ 0.05

**Caption:** G1 = group irradiated with 7 J per point; G2 = group irradiated with 9 J per point; PG = placebo group; SD = standard deviation

When comparing maximum and mean pressure and resistance before and after the intervention for all groups, the results did not indicate any statistically significant difference.

The average training frequency was 57.9%, corresponding to approximately 8.7 exercises performed a week, whereas the training approach recommends 15 exercises a week. The distribution of exercises per participant is detailed in Table 3.

**Table 3:** Mean frequency of exercise performance

Participant	Group	Exercises performed
1	G1	0
2	G2	0
3	PG	2
4	PG	18
5	G1	25
6	PG	57
7	G2	76
8	PG	76
9	PG	95
10	G1	111
11	PG	115
12	G2	120

The differences in maximum (p = 0.291) and mean (p = 0.286) pressure and resistance (p = 0.371) were not statistically significant between participants who trained and those who did not train at least 50% of the indicated frequency.

## DISCUSSION

This study verified the effects of myotherapy combined with PBM on the lips, finding no statistically significant results concerning maximum and mean lip pressure between before and after the intervention.

Contrary to this finding, published studies show that PBM with low-intensity laser causes effects on muscle tissue, improving performance, reducing fatigue, increasing strength gain, relaxing muscles, recovering tissues, and modulating inflammation^(2-7)^. However, such studies have been conducted on larger muscles, such as the biceps, quadriceps femoris^(2,3)^, tibialis anterior^(3)^, rectus femoris, and gastrocnemius^(35)^, which present anatomical characteristics distinct from those of orofacial muscles.

A randomized clinical trial whose participants had knee osteoarthritis found significant improvements in the intensity of joint pain, range of motion, physical function, and muscle strength of the quadriceps femoris and hamstrings in the group that underwent strength training plus PBM, compared to the group that only underwent strength training^(3)^. However, it is important to highlight that orofacial muscles have specific characteristics, such as fewer fiber bundles and smaller size and thickness.

The present study included participants of both sexes, aged 18 to 35 years, without changes in this musculature. The analysis indicated that the groups were homogeneous regarding age, which is important since it influences lip pressure and resistance^(12)^. Muscle mass and strength gradually decrease with age, characterized mainly by the preferential atrophy of type II fibers and necrosis of their groups^(36,37)^.

As for sex, the literature indicates that women's muscle strength is generally lower than that of males, which may be justified by the hormonal difference and the composition of muscle fibers^(38)^. Therefore, it was decided to keep only female participants in the research, since only three males had participated in all stages of the research.

A study aimed to verify the effects of tongue rotation exercises on oral functions, using maximum tongue pressure and lip closure strength in adults without alterations. Participants were divided into two different groups: in the first group, they were instructed to perform the exercise twice a day for 8 weeks; in the second group, they were instructed to perform the exercise daily (only once a day) for 12 weeks. The analysis verified that maximum tongue pressure and lip closure increased considerably, and the values ​​of men were higher than those of women at all measurement points^(39)^.

Another study with children and adolescents showed an increase in muscle strength of the lips after 16 weeks of myotherapy, performed three times a day, five times a week^(40)^. The literature indicates that myotherapy performed for at least 12 weeks with adequate frequency changes the muscle and increases lip strength and resistance parameters. The lack of changes in this research’s outcomes may be explained by the low adherence to myotherapy.

The analysis of isometric exercise performance verified low adherence – most participants did not perform the training at the requested frequency. They were asked to perform a total of 120 exercises over 8 weeks. According to the control chart, filled out individually, exercise performance varied considerably, as some participants did not perform any exercise, and one participant reported complying fully with the proposal. Five of the 12 participants (41.7%) performed less than 50% of the proposed myotherapy, which may have impacted the result, delaying muscle modification. However, there was no significant difference in pressure and resistance between those who trained up to 50% and those who trained less than 50% of the prescribed frequency, suggesting that even 8 weeks of training may not be enough to verify quantitative muscle responses of pressure and resistance in individuals without alterations.

A study found an increase in tongue muscle strength after 8 weeks of training^(41)^ in an individual with severe muscular hypotension, indicating that, in this clinical situation, changes in muscle structures occur after this period of intervention.

No statistically significant difference was found between pressure and groups. No studies comparing multiple laser applications were found in the literature. However, a study investigated the immediate effects on the orbicularis oris and found a statistically significant difference for the group that received the 7 J dose with the infrared wavelength^(12)^. Hence, it corroborates the statistical analysis of the present study, which indicated a significant difference in lip resistance after the intervention in G1, a group irradiated with the same dose and wavelength as that study. The task requested was sustained pout protrusion, which may have helped increase the resistance, considering that the participants were not subjected to exercises that recruited strength. Nevertheless, the result should be analyzed with caution because the sample was small.

In the present study, exercises were performed independently of the laser application and not subsequently. It was considered that the laser action on the tissue would last for several hours and would cover the training sessions. However, a protocol performing exercises immediately after irradiation may have better results.

It is important to highlight that the study had some limitations, such as a small sample size and the fact that the participants were young, healthy subjects with high neuroplasticity, and no changes in this musculature. Hence, it was difficult to maintain adherence in the absence of intrinsic motivation for training, leading to low frequency in training. It is also necessary to think about the number of repetitions suggested throughout the day (a total of three times), which may have been insufficient for more expressive results.

## CONCLUSION

It can be stated that PBM with low-intensity laser, with a 48-hour interval, for 8 weeks, and training aimed at the lip muscles (sustained lip protrusion exercise), with an average frequency of three executions per day, does not increase the mean maximum lip pressure. Nonetheless, lip resistance increased in the group submitted to PBM with 7 J of energy associated with myotherapy. Further studies are suggested, with greater rigor in monitoring the execution of myotherapy to verify the associated treatment, in addition to other populations, with different clinical conditions.

## Appendix A CONSORT 2010 checklist of information to include when reporting a randomised trial^*^

**Table:**

Section/Topic	Item No	Checklist item	Reported on page No
**Title and abstract**			
	1a	Identification as a randomised trial in the title	1
	1b	Structured summary of trial design, methods, results, and conclusions (for specific guidance see CONSORT for abstracts)	2
**Introduction**			
Background and objectives	2a	Scientific background and explanation of rationale	4 and 5
	2b	Specific objectives or hypotheses	4 and 5
**Methods**			
Trial design	3a	Description of trial design (such as parallel, factorial) including allocation ratio	6
	3b	Important changes to methods after trial commencement (such as eligibility criteria), with reasons	6
Participants	4a	Eligibility criteria for participants	6
	4b	Settings and locations where the data were collected	6
Interventions	5	The interventions for each group with sufficient details to allow replication, including how and when they were actually administered	7 to 11
Outcomes	6a	Completely defined pre-specified primary and secondary outcome measures, including how and when they were assessed	11
	6b	Any changes to trial outcomes after the trial commenced, with reasons	-
Sample size	7a	How sample size was determined	11
	7b	When applicable, explanation of any interim analyses and stopping guidelines	-
Randomisation:			
Sequence generation	8a	Method used to generate the random allocation sequence	6
	8b	Type of randomisation; details of any restriction (such as blocking and block size)	6
Allocation concealment mechanism	9	Mechanism used to implement the random allocation sequence (such as sequentially numbered containers), describing any steps taken to conceal the sequence until interventions were assigned	6
Implementation	10	Who generated the random allocation sequence, who enrolled participants, and who assigned participants to interventions	6
Blinding	11a	If done, who was blinded after assignment to interventions (for example, participants, care providers, those assessing outcomes) and how	6-7
	11b	If relevant, description of the similarity of interventions	
Statistical methods	12a	Statistical methods used to compare groups for primary and secondary outcomes	11
	12b	Methods for additional analyses, such as subgroup analyses and adjusted analyses	11
**Results**			
Participant flow (a diagram is strongly recommended)	13a	For each group, the numbers of participants who were randomly assigned, received intended treatment, and were analysed for the primary outcome	12-13
	13b	For each group, losses and exclusions after randomisation, together with reasons	Not applicable
Recruitment	14a	Dates defining the periods of recruitment and follow-up	6
	14b	Why the trial ended or was stopped	6
Baseline data	15	A table showing baseline demographic and clinical characteristics for each group	Not applicable
Numbers analysed	16	For each group, number of participants (denominator) included in each analysis and whether the analysis was by original assigned groups	12-13
Outcomes and estimation	17a	m	
	17b	For binary outcomes, presentation of both absolute and relative effect sizes is recommended	Not applicable
Ancillary analyses	18	Results of any other analyses performed, including subgroup analyses and adjusted analyses, distinguishing pre-specified from exploratory	12-13
Harms	19	All important harms or unintended effects in each group (for specific guidance see CONSORT for harms)	
**Discussion**			
Limitations	20	Trial limitations, addressing sources of potential bias, imprecision, and, if relevant, multiplicity of analyses	16
Generalisability	21	Generalisability (external validity, applicability) of the trial findings	
Interpretation	22	Interpretation consistent with results, balancing benefits and harms, and considering other relevant evidence	
**Other information**			
Registration	23	Registration number and name of trial registry	RBR-6pygc5m
Protocol	24	Where the full trial protocol can be accessed, if available	
Funding	25	Sources of funding and other support (such as supply of drugs), role of funders	

* We strongly recommend reading this statement in conjunction with the CONSORT 2010 Explanation and Elaboration for important clarifications on all the items. If relevant, we also recommend reading CONSORT extensions for cluster randomised trials, non-inferiority and equivalence trials, non-pharmacological treatments, herbal interventions, and pragmatic trials. Additional extensions are forthcoming: for those and for up to date references relevant to this checklist, see SPIRIT–CONSORT website^(42)^

## Appendix B Control chart

**Table:**

	Repetition - morning	Repetition - afternoon	Repetition –evening
**Monday**			
**Tuesday**			
**Wednesday**			
**Thursday**			
**Friday**			
