# Supplementary material for: Effects of myotherapy combined with photobiomodulation on the lips: a randomized clinical trial
Source: Codas. 2025 Aug 8;37(4):e20240144. doi: 10.1590/2317-1782/e20240144en (PMC12419456; doi:10.1590/2317-1782/e20240144en)
Supplement: Translation

# Translation: Efeitos da mioterapia combinada com a fotobiomodulação sobre os lábios: ensaio clínico randomizado

**DOI:** 10.1590/2317-1782/e20240144pt

**Authors:** Mariana Rodrigues Batista, Andréa Rodrigues Motta, Renata Maria Moreira Moraes Furlan

**Affiliations:** 1 Programa de Pós-graduação em Ciências Fonoaudiológicas, Universidade Federal de Minas Gerais – UFMG - Belo Horizonte (MG), Brasil.; 2 Departamento de Fonoaudiologia, Faculdade de Medicina, Universidade Federal de Minas Gerais – UFMG - Belo Horizonte (MG), Brasil.

**Keywords:** Lábio, Força Muscular, Tono Muscular, Terapia a Laser, Terapia Miofuncional

## Abstract

**Objetivo:**

comparar a pressão máxima, média e a resistência labial de adultos hígidos submetidos à mioterapia associada à fotobiomodulação, com diferentes doses.

**Método:**

foi realizado um ensaio clínico randomizado, duplo-cego. A amostra, não probabilística, composta por 12 indivíduos, com média de idade de 21,8 anos, alocados aleatoriamente em três grupos distintos para a intervenção com a fotobiomodulação. Os exercícios foram os mesmos para todos os participantes. No grupo 1 (G1), os participantes foram submetidos à fotobiomodulação com dose de 7 J por ponto; no grupo 2 (G2), os participantes foram irradiados com 9 J por ponto; e, no grupo placebo (GP), os participantes foram submetidos aos mesmos procedimentos dos demais grupos, entretanto o equipamento foi ligado, mas não ativado (placebo). O comprimento de onda utilizado foi o infravermelho. Os participantes receberam a fotobiomodulação três vezes por semana, com intervalo de 48 horas, por oito semanas. A mioterapia foi realizada em domicílio pelos participantes, três vezes ao dia, cinco vezes por semana, por oito semanas. Foi realizada a avaliação clínica dos lábios, avaliação da pressão máxima, média e resistência labial, por meio do Iowa Oral Performance Instrument (IOPI), feitas pré e pós-intervenção.

**Resultados:**

não houve resultados que demonstrassem o aumento da pressão máxima ou média dos lábios, entretanto houve aumento da resistência labial no G1.

**Conclusão:**

a fotobiomodulação associada à mioterapia, com os parâmetros e a metodologia realizada neste estudo, não resultou em aumento da pressão labial, mas promoveu aumento da resistência no grupo exposto a 7J por ponto.

## INTRODUÇÃO

O termo “laser”, que vem do inglês, é traduzido para o português como amplificação de luz por emissão estimulada de radiação^(1)^. A fotobiomodulação (FBM) com o laser de baixa intensidade é um método de tratamento que não utiliza medicamentos, é indolor, não invasivo e não apresenta efeitos colaterais^(1)^. Os seus benefícios incluem melhor desempenho muscular, retardo da fadiga, relaxamento muscular, aumento do ganho de força, alívio da dor, redução da inflamação e estímulo à regeneração tecidual^(1-7)^.

Esses resultados podem ser atribuídos ao fato de que as células humanas são unidades funcionais que podem ser ativadas por efeitos fotoquímicos, fotofísicos e fotobiológicos quando os tecidos são irradiados^(8)^. O laser de baixa intensidade é um tipo de radiação não-ionizante que proporciona a estimulação celular, sem efeito destrutivo^(9)^. Ao entrar no tecido biológico, a luz consegue transferir sua energia para aquele meio. A absorção depende da ação dos cromóforos, que são moléculas presentes, principalmente nas mitocôndrias e que convertem a energia luminosa em energia química durante o processo de metabolismo celular^(10)^. Por meio de reações enzimáticas, o laser promove o aumento da quantidade de adenosina trifosfato (ATP) dentro das células^(10)^.

A FBM tem sido associada ao treino em diferentes musculaturas, especialmente grandes grupos. Para grupos musculares menores como os orofaciais, existem poucas evidências para embasar a utilização do recurso^(11-13)^. Ainda assim, fonoaudiólogos reportam estar aplicando a FBM na sua prática clínica^(14)^.

A mioterapia, abordagem terapêutica presente na prática clínica na área de Motricidade Orofacial, visa à modificação muscular por meio da realização de exercícios direcionados à musculatura que se pretende estimular^(15)^. Sabendo que a atividade muscular requer uma grande quantidade de energia, tanto para a contração quanto para o relaxamento e a manutenção do tônus corporal^(16)^ e a fotobiomodulação com o laser de baixa intensidade promove aumento de ATP no interior das células^(9)^, acredita-se que a FBM tem potencial para acelerar o processo de modificação muscular e consequentemente otimizar o tempo de terapia.

Um estudo investigou os efeitos da fotobiomodulação sobre a musculatura anterior na coxa e verificou que, os participantes que foram irradiados antes do treinamento muscular apresentaram aumento significativo da contração voluntária máxima (CVM), além do efeito positivo no teste de repetição máxima^(5)^.

No que se refere aos efeitos da FBM nos lábios, Mouffron et al.^(12)^ verificaram aumento da pressão labial imediatamente após irradiação com laser de comprimento de onda infravermelho e 7 J de energia, enquanto Batista et al.^(13)^ não encontraram alteração da fadiga eletromiográfica do orbicular da boca pela irradiação com dose de 4 J. Não foram encontrados na literatura estudos com mais de uma sessão de fotobiomodulação ou estudos que fizessem a combinação do treino muscular com a irradiação. Acredita-se que quanto maior a dose, maior será o efeito fotoquímico e, como consequência, melhor desempenho muscular.

Diante disso, este trabalho tem por objetivo verificar se a fotobiomodulação associada à mioterapia potencializa o ganho de pressão e de resistência dos lábios em adultos hígidos.

## MÉTODO

Este estudo trata-se de um ensaio clínico randomizado, duplo-cego, realizado na Universidade Vale do Rio Doce (UNIVALE). O estudo foi aprovado pelo Comitê de Ética em Pesquisa da instituição (Parecer 6.237.793), registrado na plataforma de Registro Brasileiro de Ensaios Clínicos (ReBEC) (RBR-6pygc5m) e desenvolvido seguindo Checklist Consort^(17)^ (Apêndice A). Todos os participantes assinaram o Termo de Consentimento Livre e Esclarecido. O cegamento deu-se da seguinte maneira: a pesquisadora que realizou a avaliação dos participantes não sabia qual grupo de intervenção cada participante pertencia. A pesquisadora que realizou a intervenção não sabia do resultado da avaliação e nem mesmo os participantes sabiam com qual dose estavam sendo irradiados. A coleta de dados foi realizada entre o período de agosto a dezembro de 2023. Esses são os dados preliminares de uma pesquisa que ainda está em andamento.

A amostra selecionada foi não probabilística, composta por 12 indivíduos, sendo alunos da UNIVALE, em Governador Valadares, com média de idade de 21,8 anos (DP= 4,0; mínimo=18; máximo=28), sendo todos do sexo feminino. O recrutamento foi realizado por meio de anúncios durante as aulas e divulgação em redes sociais.

Os participantes foram divididos, de maneira randomizada, por sorteio, em três grupos: grupo 1 (G1), grupo 2 (G2) e grupo placebo (GP). Para tanto, os participantes pegavam um papel contendo um número dentro de uma caixa.

Foram incluídos indivíduos hígidos com idade entre 18 e 35 anos, que não haviam feito uso de medicação miorrelaxante e/ou anti-inflamatória^(18)^ no período de 48 horas que antecedeu a coleta dos dados, que não estavam realizando terapia miofuncional orofacial e que apresentavam capacidade de realização do exercício de protrusão labial sustentada. Foram excluídos os participantes que abandonaram a pesquisa, não retornando para reavaliação; participantes que apresentaram contraindicações para fototerapia, conforme manual dos fabricantes do equipamento: fotossensibilidade, gravidez, glaucoma, lesão sem diagnóstico sobre a área a ser irradiada ou próxima a ela, infecção no local da aplicação, histórico de câncer, uso de marcapasso ou outro implante eletrônico.

### Procedimentos

Os procedimentos de avaliação e fotobiomodulação foram realizados no ambulatório de Motricidade Orofacial das Clínicas Integradas da Universidade Vale do Rio Doce. Os procedimentos foram divididos em três etapas: uma sessão de avaliação inicial (etapa 1), oito semanas de tratamento individual (etapa 2), uma sessão de reavaliação final (etapa 3), exemplificado na Figura 1.

**Figura 1:** Procedimentos de avaliação, intervenção e reavaliação

#### Etapa 1 - Avaliação

Na etapa 1, começou-se com uma sessão de instrução sobre a execução correta do exercício, seguida pela verificação da capacidade do participante para realizá-lo. Logo após, foi feita a avaliação inicial, em que os participantes foram submetidos aos procedimentos de avaliação clínica dos lábios, avaliação da pressão labial e avaliação da resistência labial.

Os processos de avaliação e FBM com o laser de baixa intensidade foram conduzidos por pesquisadores diferentes, garantindo-se, assim, que o avaliador não tivesse conhecimento sobre a alocação dos participantes em grupos específicos.

Para garantir uma análise imparcial dos dados, todos os arquivos de avaliação foram numerados e os nomes dos participantes removidos, evitando-se, assim, a identificação dos grupos aos quais pertenciam durante a análise (se pré ou pós-treinamento).

##### Avaliação clínica dos lábios

Foi orientado que o participante permanecesse sentado em uma cadeira, em flexão de 90º entre quadril, joelho e tornozelos, postura ereta, guiada pelo Plano de Frankfurt. Foi realizada a avaliação intra e extraoral e de mobilidade dos lábios de acordo com o preconizado no protocolo MBGR – Avaliação Miofuncional Orofacial^(19)^ para caracterização da amostra.

##### Avaliação de pressão labial

Para obter-se os valores de pressão labial de cada participante, foi utilizado o IOPI (*Iowa Oral Performance Instrument)*. O bulbo do IOPI foi colocado entre duas espátulas de madeira e envolvido num plástico filme^(12,20,21)^. Os participantes foram solicitados a pressionar o bulbo com máxima força possível, com movimento de preensão labial, durante 2 segundos. Foram realizadas três repetições, com intervalo de trinta segundos entre elas e o valor de pico máximo foi considerado a pressão máxima labial. Também foi registrada a pressão média, que se refere à média aritmética do pico de pressão obtido em cada uma das três repetições.

##### Avaliação da resistência labial

Foi utilizado o IOPI para obter os valores de resistência labial, com o bulbo entre duas espátulas de madeira e envolvido por plástico filme. Os participantes foram solicitados a pressionar o bulbo, mantendo 50% da pressão máxima pelo maior tempo possível^(21)^. Houve feedback visual em tempo real para indicar o valor de pressão que deveria ser mantido. Foi considerada como resistência labial, o tempo, em segundos, em que o participante foi capaz de manter o bulbo pressionado.

#### Etapa 2 - Fotobiomodulação e Mioterapia

Nesta fase, os participantes foram distribuídos aleatoriamente em três grupos por meio de sorteio. Os participantes não sabiam em qual grupo eles estavam alocados e nem mesmo qual intervenção receberiam. As pesquisadoras que realizaram a análise dos dados, também, não tiveram acesso a qual tipo de intervenção cada participante recebeu, mantendo o cegamento até o final da pesquisa.

- Grupo 1 (G1): submetido a mioterapia e FBM com comprimento de onda infravermelho e dose de 7 J por ponto totalizando 42 J;
- Grupo 2 (G2): submetido a mioterapia e FBM com comprimento de onda infravermelho e dose de 9 J por ponto totalizando 54 J;
- Grupo Placebo (GP): submetido a mioterapia e ao procedimento placebo para FBM (o equipamento foi posicionado e ligado, mas não houve emissão de luz).

A irradiação deu-se por contato com a pele, sendo dois pontos na porção superior do músculo orbicular da boca, dois pontos na porção inferior e um ponto em cada comissura labial, conforme demonstrado na Figura 2.

**Figura 2:** Marcação dos pontos de aplicação do laser

##### Mioterapia

A mioterapia foi realizada em oito semanas de tratamento com execução domiciliar de três vezes por dia em cinco dias por semana.

A intervenção terapêutica foi escolhida com base nos achados da literatura, quanto ao tipo de exercício, repetições, frequência de treino e duração do tratamento. Um estudo propôs investigar e comparar a atividade elétrica do músculo orbicular da boca em diferentes exercícios de contração máxima voluntária isométrica. Como resultado, verificou-se que o exercício de protrusão labial com fechamento apresentou maior atividade elétrica no músculo orbicular da boca^(22)^.

O participante foi orientado a realizar três repetições^(23,24)^ do exercício isométrico de protrusão labial com fechamento^(22)^, três vezes ao dia^(24,25)^, cinco dias na semana^(26,27)^, durante um período de oito semanas^(24,28)^. O treino proposto teve carga progressiva, sendo o tempo de sustentação do exercício aumentado progressivamente a cada semana, conforme o Quadro 1.

**Quadro 1:** Número de repetições e tempo de contração do exercício de protrusão labial realizado pelos participantes em cada semana de tratamento

Semana	Repetições	Tempo de contração (s)
1	3	5
2	3	10
3	3	15
4	3	20
5	3	25
6	3	30
7	3	35
8	3	40

Cada participante executou o exercício, seguindo um passo a passo e preencheu um quadro controle (Apêndice B) enviado por e-mail e disponibilizado no Microsoft Word Online, conforme a sua execução, incluindo dados como a frequência de realização por dia. O participante assinalou com “X”, informando a realização do exercício naquele turno, do dia em questão.

##### Fotobiomodulação

Após a avaliação inicial, foram realizadas as aplicações do laser, com equipamento da marca MMO®, modelo Laser Duo e 100 mW de potência. No Quadro 2, constam os parâmetros da irradiação.

**Quadro 2:** Parâmetros da irradiação

Parâmetros de irradiação	Valores
Comprimento de onda	808 nm (infravermelho)
Modo de operação	Contínuo
Potência	100 mW
Diâmetro do spot de saída	1,95 mm
Área do spot de saída	0,03 cm^3^
Densidade de potência	3,3 W/cm^2^
Energia por ponto	7 J / 9 J
Densidade de energia (fluência) por ponto	133,3 J/cm^3^
Tempo de aplicação por ponto	70 s / 90 s

Foi escolhido o laser de comprimento de onda de 808 nm (infravermelho)^(12,13)^ para irradiação, tendo como parâmetros dosimétricos: 7 J^(29-32)^ e 9 J por ponto de aplicação. Um ensaio clínico randomizado obteve como efeitos imediatos a modificação da pressão labial e melhora no desempenho no músculo dos lábios^(12)^, após a fotobiomodulação realizada no comprimento de onda de 808 nm, com dose de 7 J.

Outro estudo, que investigou os efeitos imediatos da fotobiomodulação sobre a fadiga eletromiográfica do músculo orbicular da boca, utilizou a dose de 4 J por ponto de aplicação, sendo dois pontos na porção superior e dois pontos na porção inferior e não encontrou diferenças estatisticamente significativas, ao comparar os resultados pré e pós irradiação^(13)^.

Uma revisão integrativa da literatura^(33)^ apontou que as dosimetrias mais utilizadas, por ponto, foram 7 J e 30 J. Considerando as particularidades do orbicular da boca, sobretudo em relação à espessura e extensão, optou-se por não realizar a aplicação de 30 J por ponto, mas sim 9 J, que é a máxima dose disponível para aplicação única (direta) no equipamento. Foram irradiados seis pontos: um ponto em cada comissura labial, dois pontos na porção superior do músculo orbicular da boca, dois pontos na porção inferior do músculo.

A FBM foi aplicada três vezes por semana, por pesquisadoras previamente treinadas, no Ambulatório de Motricidade Orofacial das Clínicas Integradas da UNIVALE mantendo intervalo mínimo de 48 horas entre as aplicações^(34)^. Durante a irradiação os participantes encontravam-se sentados, com costas e pés apoiados, utilizando os óculos de proteção. Para garantir a segurança dos aplicadores e dos participantes, foram seguidas as recomendações do fabricante do equipamento e as normas de segurança estabelecidas pela Agência Nacional de Vigilância Sanitária (ANVISA) para equipamentos FBM.

Para aplicação do procedimento placebo o equipamento foi ligado, mas não houve emissão de luz. O equipamento foi posicionado com contato sobre os seis pontos de aplicação por tempo entre 70 e 90 s, equivalente às doses de 7 e 9 J, respectivamente, caso estivesse sendo irradiado.

#### Etapa 3 - Reavaliação

Cada participante foi reavaliado, pelo mesmo avaliador inicial, por meio dos mesmos procedimentos utilizados na avaliação inicial na semana seguinte após a última sessão de tratamento.

### Análise dos dados

Constituíram variáveis resposta (desfechos) da presente pesquisa a pressão média e máxima e a resistência dos lábios. Primeiramente, foi aplicado o teste Shapiro-Wilk, em que se verificou que os dados não apresentaram distribuição normal. A homogeneidade quanto à idade, pressão máxima, pressão média e resistência, mensuradas antes da intervenção foi verificada por meio do teste de Kruskal-Wallis.

O teste de Wilcoxon - teste não-paramétrico para comparação de duas amostras pareadas - foi utilizado para as comparações da pressão máxima média e da resistência entre os momentos pré e pós-intervenção.

A frequência de realização dos exercícios pelos participantes ao longo das oito semanas foi calculada com base nas respostas dos questionários e analisada de forma descritiva pela frequência relativa, de acordo com o apresentado na Figura 3.

**Figura 3:** Frequência da realização de exercícios

As diferenças de pressão e de resistência labial do momento pós para o momento pré-intervenção foram comparadas entre indivíduos que realizaram o treino em até 50% e aqueles que realizaram mais do que 50% da frequência estipulada por meio do teste de Kruskal-Wallis.

Adotou-se nível de significância de 5% em todas as comparações.

## RESULTADOS

Inicialmente, 29 participantes foram avaliados e incluídos para a coleta de dados. Entretanto, somente 12 participantes permaneceram até a etapa de reavaliação final. Quatorze participantes foram excluídos após abandonarem a pesquisa. Desses, cinco participantes pertenciam ao G1, três participantes pertenciam ao grupo 2, três participantes pertenciam ao GP e três participantes não chegaram a receber nenhuma intervenção e, por isso, não haviam sido alocados em nenhum grupo. Para a análise de dados, optou-se por excluir mais três participantes, os quais eram do sexo masculino, uma vez que não foi possível obter uma amostra equilibrada quanto ao sexo.

Os resultados indicaram que os grupos são homogêneos em relação à idade (p=0,603), pressão máxima pré-intervenção (p=0,207) e pressão média pré-intervenção (p= 0,149) e resistência (p=0,059).

A Tabela 1 apresenta a comparação da pressão máxima, média e resistência dos lábios, antes e após a intervenção em cada grupo.

**Tabela 1:** Pressão máxima (kPa), média (kPa) e resistência dos lábios antes e após a aplicação do laser

Grupo		Pré	Pós	Pré	Pós	Pré	Pós
		(Máxima)	(Máxima)	(Média)	(Média)	(Resistência)	(Resistência)
G1	Média	12,0	7,0	10,7	6	22,7	211,7
	DP	6,6	1,73	5,7	1,7	2,3	287,9
	Mediana	11,0	6	9	5	24	54
	Mínimo	6	6	6	5	20	37
	Máximo	19	9	17	8	24	544
	Valor de p	0,246		0,121		0,0463*	
G2	Média	7,6	9	6,7	8	91	53
	DP	0,6	2	0,6	2	38,5	38,5
	Mediana	8	9	7	7	90	54
	Mínimo	7	7	6	6	53	14
	Máximo	8	11	7	10	130	91
	Valor de p	0,369		0,368		0,513	
GP	Média	6,6	7,4	5,6	6,8	65,4	42,8
	DP	1,3	3,5	0,9	3,2	23,5	29,2
	Mediana	6	7	5	7	56	28
	Mínimo	6	4	5	4	47	17
	Máximo	9	13	7	12	104	76
	Valor de p	0,914		0,747		0,347	

Teste de Wilcoxon. Nível de significância de 5%

* Valor de p≤0,05

**Legenda:** G1 = grupo irradiado com 7 J por ponto; G2 = grupo irradiado com 9 J por ponto; GP = grupo placebo; DP = desvio padrão

A análise comparativa da pressão máxima e média dos lábios, antes e após o participante ser submetido a FBM, não indicou diferença com relevância estatística.

A análise comparativa sobre a resistência entre os momentos pré e pós-intervenção, indicou diferença com relevância estatística para o G1, com aumento da resistência após a intervenção.

A Tabela 2 apresenta a comparação entre os grupos para a diferença de pressão máxima, média e resistência, calculada pelo valor no momento pós menos o valor no pré-intervenção.

**Tabela 2:** Diferença entre pressão máxima, média e resistência entre todos os grupos

Grupo	Pressão máxima		Pressão média		Resistência	
	média	DP	Média	DP	Média	DP
G1	-5,0	5,0	-4,7	4,0	189	286,8
G2	1,3	2,5	1,3	2,0	-38	77,0
GP	0,8	4,4	1,2	3,8	-22,6	38,8
Valor de p*	0,282		0,186		0,234	

Teste de Kruskal-Wallis. Nível de significância de 5%. Valores negativos significam que o valor no pós foi menor que no pré-intervenção

* Valor de p≤0,05

**Legenda:** G1 = grupo irradiado com 7 J por ponto; G2 = grupo irradiado com 9 J por ponto; GP = grupo placebo; DP = desvio padrão

Na comparação da pressão máxima, média e resistência, pré e pós, para todos os grupos, os resultados obtidos não indicaram diferença estatisticamente significante.

A frequência média de treino foi de 57,9%, correspondendo a cerca de 8,7 execuções de exercício por semana, enquanto o treinamento proposto preconizava 15 execuções de exercício semanalmente, sendo a distribuição dos exercícios realizados por cada participante detalhada na Tabela 3.

**Tabela 3:** Frequência média da realização de exercícios

Participante	Grupo	Exercícios Realizados
1	G1	0
2	G2	0
3	GP	2
4	GP	18
5	G1	25
6	GP	57
7	G2	76
8	GP	76
9	GP	95
10	G1	111
11	GP	115
12	G2	120

Não houve diferença com significância estatística para as diferenças de pressões máxima (p= 0,291) e média (p=0,286), bem como para a diferença de resistência (p=0,371) entre os participantes que treinaram e os que não realizaram o treino em pelo menos 50% da frequência indicada.

## DISCUSSÃO

O presente estudo verificou os efeitos da mioterapia combinada com a FBM sobre os lábios, não sendo observados resultados com significância estatística entre os momentos pré e pós-intervenção, sobre as variáveis pressão máxima e média labial.

Contrários a este achado, estudos publicados evidenciam que a FBM com o laser de baixa intensidade provoca efeitos sobre o tecido muscular, promovendo melhor desempenho, redução da fadiga, aumento do ganho de força, relaxamento muscular, recuperação tecidual e modulação da inflamação^(2-7)^ No entanto, tais estudos foram realizados com músculos maiores, tais como bíceps e quadríceps femoral^(2,3)^, músculo tibial anterior^(3)^, reto femural e gastrocnêmio^(35)^, tendo características anatômicas diferentes dos músculos orofaciais.

Um ensaio clínico randomizado com participantes portadores de osteoartrite de joelho verificou melhora significativa na intensidade da dor articular, amplitude do movimento, função física e força muscular do quadríceps femoral e isquiotibiais no grupo que foi submetido ao treinamento de força acrescido da fotobiomodulação comparado ao grupo que somente foi submetido ao treinamento de força^(3)^. Entretanto, é necessário destacar que os músculos orofaciais possuem características específicas, tais como menor quantidade de feixes de fibras, menor tamanho e espessura.

Foram incluídos, na presente pesquisa, participantes de ambos os sexos, com idade entre 18 e 35 anos e sem alterações nesta musculatura. Em relação à idade, a análise indicou que os grupos foram homogêneos, o que é importante, visto que a pressão e a resistência labial sofrem a influência dessa variável^(12)^. À medida que o indivíduo envelhece, há uma redução gradual na massa e na força muscular, caracterizada principalmente pela atrofia preferencial das fibras do tipo II e necrose dos seus agrupamentos^(36,37)^.

Quanto ao sexo, a literatura aponta que, em geral, a força muscular das mulheres é menor, quando comparada à força dos indivíduos do sexo masculino, podendo ser justificada pela diferença hormonal e da composição de fibras musculares^(38)^. Dessa forma, optou-se por manter apenas participantes do sexo feminino na pesquisa, visto que apenas três indivíduos do sexo masculino haviam participado de todas as etapas da pesquisa.

Uma pesquisa realizada objetivou-se a estudar os efeitos do exercício de rotação de língua sobre as funções orais, utilizando a medição de pressão máxima lingual e da força de fechamento labial em adultos sem alterações. Os participantes foram alocados em dois grupos diferentes: no primeiro grupo foram orientados a realizarem o exercício duas vezes ao dia, durante oito semanas; no segundo grupo os participantes foram orientados a realizarem o exercício diariamente (somente uma vez por dia), durante um período de 12 semanas. Após a análise foi possível verificar que a pressão máxima de língua e de vedamento labial aumentou consideravelmente e os valores dos homens foram maiores do que os das mulheres em todos os pontos de medição^(39)^.

Outro estudo, feito com crianças e adolescentes, apontou o aumento da força muscular dos lábios após 16 semanas de mioterapia, realizada na frequência de três vezes ao dia e cinco vezes por semana^(40)^. Diante da literatura é possível verificar que a mioterapia realizada em período mínimo de 12 semanas, com frequência adequada, promove modificação muscular e aumento dos parâmetros de força e resistência labial e possivelmente a ausência de modificação dos desfechos avaliados nesta pesquisa tem explicação na baixa adesão à mioterapia.

Ao verificar a realização do exercício isométrico, foi possível constatar que houve baixa adesão, tendo os participantes em sua maioria, não realizado o treino na frequência solicitada. Os participantes foram solicitados a realizar um total de 120 exercícios, distribuídos ao longo de oito semanas. De acordo com o quadro-controle, preenchido individualmente, a realização dos exercícios apresentou grande variação, possuindo participantes que não computaram nenhuma execução do exercício e participante que informou que cumpriu integralmente o proposto. Do total de 12 participantes, cinco (41,7%) realizaram menos de 50% da mioterapia proposta, o que pode ter impactado no resultado, retardando a modificação muscular. No entanto, não houve diferença significativa para a diferença de pressão e de resistência entre quem treinou até 50% e aqueles que treinaram menos que 50% do previsto, sugerindo que mesmo oito semanas de treino pode não ser um tempo suficiente para verificar respostas musculares quantitativas de pressão e de resistência em indivíduos sem alteração.

Um estudo encontrou aumento na força muscular da língua após oito semanas de treinamento^(41)^, em indivíduo com hipotensão muscular severa, indicando que, nessa situação clínica, ocorre modificação das estruturas musculares após esse tempo de intervenção.

Ao comparar a pressão e entre grupos, não foi encontrada diferença estatística com significância. Ao pesquisar a literatura, não foram encontrados estudos com a comparação de múltiplas aplicações de laser, mas um estudo que investigou os efeitos imediatos para o orbicular da boca verificou diferença com significância estatística para o grupo que recebeu a dose de 7 J, com o comprimento de onda infravermelho^(12)^, corroborando a análise estatística deste estudo, que apontou diferença com relevância sobre a resistência labial após a intervenção no G1, grupo irradiado pela mesma dose e comprimento de onda do estudo citado. A tarefa solicitada foi bico protruso sustentado, o que pode ter favorecido o aumento da resistência, tendo em vista que os participantes não foram submetidos a exercício que recrutasse a força. Apesar disso, o resultado deve ser analisado com cautela, devido a amostra ser reduzida.

Na presente pesquisa, o momento da execução do exercício aconteceu de forma independente da aplicação do laser e não subsequente. Considerou-se que a ação do laser no tecido perduraria por várias horas e abrangeria os treinos. É possível que um protocolo de realização de exercícios imediatamente após irradiação tenha melhores resultados.

É necessário destacar que o estudo apresentou algumas limitações, tamanho reduzido da amostra e o fato de que os participantes eram sujeitos jovens, saudáveis, com alta neuroplasticidade, além de não apresentarem alterações nesta musculatura, sendo difícil manter a adesão na ausência de motivação intrínseca para o treino, promovendo baixa frequência na realização dos treinos. Ainda é necessário pensar a respeito da quantidade de repetições sugeridas ao longo do dia (total de 3 vezes), podendo ter sido insuficiente para resultados mais expressivos.

## CONCLUSÃO

Pode-se afirmar que a fotobiomodulação com o laser de baixa intensidade, com intervalo de 48 horas, por um período de oito semanas, e treino direcionado à musculatura labial (exercício de protrusão labial sustentada), com frequência média de 3 execuções por dia, não promove o aumento da pressão máxima, média dos lábios. Houve aumento da resistência labial no grupo submetido à fotobiomodulação com 7 J de energia associada à mioterapia. Sugere-se a realização de outros estudos, com maior rigor no acompanhamento da execução da mioterapia para verificar o tratamento associado, além de outras populações, com diferentes condições clínicas.

## Apêndice A CONSORT 2010 checklist of information to include when reporting a randomised trial*

**Table:**

Section/Topic	Item No	Checklist item	Reported on page No
**Title and abstract**			
	1a	Identification as a randomised trial in the title	1
	1b	Structured summary of trial design, methods, results, and conclusions (for specific guidance see CONSORT for abstracts)	2
**Introduction**			
Background and objectives	2a	Scientific background and explanation of rationale	4 e 5
	2b	Specific objectives or hypotheses	4 e 5
**Methods**			
Trial design	3a	Description of trial design (such as parallel, factorial) including allocation ratio	6
	3b	Important changes to methods after trial commencement (such as eligibility criteria), with reasons	6
Participants	4a	Eligibility criteria for participants	6
	4b	Settings and locations where the data were collected	6
Interventions	5	The interventions for each group with sufficient details to allow replication, including how and when they were actually administered	7 a 11
Outcomes	6a	Completely defined pre-specified primary and secondary outcome measures, including how and when they were assessed	11
	6b	Any changes to trial outcomes after the trial commenced, with reasons	-
Sample size	7a	How sample size was determined	11
	7b	When applicable, explanation of any interim analyses and stopping guidelines	-
Randomisation:			
Sequence generation	8a	Method used to generate the random allocation sequence	6
	8b	Type of randomisation; details of any restriction (such as blocking and block size)	6
Allocation concealment mechanism	9	Mechanism used to implement the random allocation sequence (such as sequentially numbered containers), describing any steps taken to conceal the sequence until interventions were assigned	6
Implementation	10	Who generated the random allocation sequence, who enrolled participants, and who assigned participants to interventions	6
Blinding	11a	If done, who was blinded after assignment to interventions (for example, participants, care providers, those assessing outcomes) and how	6-7
	11b	If relevant, description of the similarity of interventions	
Statistical methods	12a	Statistical methods used to compare groups for primary and secondary outcomes	11
	12b	Methods for additional analyses, such as subgroup analyses and adjusted analyses	11
**Results**			
Participant flow (a diagram is strongly recommended)	13a	For each group, the numbers of participants who were randomly assigned, received intended treatment, and were analysed for the primary outcome	12-13
	13b	For each group, losses and exclusions after randomisation, together with reasons	Not applicable
Recruitment	14a	Dates defining the periods of recruitment and follow-up	6
	14b	Why the trial ended or was stopped	6
Baseline data	15	A table showing baseline demographic and clinical characteristics for each group	Not applicable
Numbers analysed	16	For each group, number of participants (denominator) included in each analysis and whether the analysis was by original assigned groups	12-13
Outcomes and estimation	17a	m	
	17b	For binary outcomes, presentation of both absolute and relative effect sizes is recommended	Not applicable
Ancillary analyses	18	Results of any other analyses performed, including subgroup analyses and adjusted analyses, distinguishing pre-specified from exploratory	12-13
Harms	19	All important harms or unintended effects in each group (for specific guidance see CONSORT for harms)	
**Discussion**			
Limitations	20	Trial limitations, addressing sources of potential bias, imprecision, and, if relevant, multiplicity of analyses	16
Generalisability	21	Generalisability (external validity, applicability) of the trial findings	
Interpretation	22	Interpretation consistent with results, balancing benefits and harms, and considering other relevant evidence	
**Other information**			
Registration	23	Registration number and name of trial registry	RBR-6pygc5m
Protocol	24	Where the full trial protocol can be accessed, if available	
Funding	25	Sources of funding and other support (such as supply of drugs), role of funders	

* We strongly recommend reading this statement in conjunction with the CONSORT 2010 Explanation and Elaboration for important clarifications on all the items. If relevant, we also recommend reading CONSORT extensions for cluster randomised trials, non-inferiority and equivalence trials, non-pharmacological treatments, herbal interventions, and pragmatic trials. Additional extensions are forthcoming: for those and for up to date references relevant to this checklist, see SPIRIT–CONSORT website^(42)^

## Apêndice B Quadro controle

**Table:**

	Repetição - manhã	Repetição - tarde	Repetição – noite
**Segunda**			
**Terça**			
**Quarta**			
**Quinta**			
**Sexta**
